# Evolutionary perspectives on wildlife disease: concepts and applications

**DOI:** 10.1111/eva.12179

**Published:** 2014-08-27

**Authors:** Eric Vander Wal, Dany Garant, Fanie Pelletier

**Affiliations:** Département de biologie, Université de SherbrookeSherbrooke, QC, Canada

**Keywords:** applied evolution, conservation, epidemiology, host–pathogen interactions, wildlife management, zoonosis

## Abstract

Wildlife disease has the potential to cause significant ecological, socioeconomic, and health impacts. As a result, all tools available need to be employed when host–pathogen dynamics merit conservation or management interventions. Evolutionary principles, such as evolutionary history, phenotypic and genetic variation, and selection, have the potential to unravel many of the complex ecological realities of infectious disease in the wild. Despite this, their application to wildlife disease ecology and management remains in its infancy. In this article, we outline the impetus behind applying evolutionary principles to disease ecology and management issues in the wild. We then introduce articles from this special issue on *Evolutionary Perspectives on Wildlife Disease: Concepts and Applications,* outlining how each is exemplar of a practical wildlife disease challenge that can be enlightened by applied evolution. Ultimately, we aim to bring new insights to wildlife disease ecology and its management using tools and techniques commonly employed in evolutionary ecology.

## Introduction

Parasites and pathogens have significant implications for wildlife and can have severe socioeconomic consequences (Daszak et al. 2000). For example, 60% of emerging infectious diseases are zoonotic; the majority of which originate in wildlife (Jones et al. 2008a). A trend likely to persist because of the increased global movements of species by humans (Olden et al. 2004). Moreover, biodiversity loss can increase rates of pathogens transmission (Keesing et al. 2010; Salkeld et al. 2013). Although disease has rarely been implicated in causing endangerment (Smith et al. 2006), it has the potential to cause extirpation and extinction, particularly in small populations and for pathogens whose transmission is independent of population density (De Castro and Bolker 2005; McCallum 2012). In cases where socioeconomic, health, or conservation concerns exist, management remains an important tool to protect populations (human and nonhuman) from the effects of wildlife disease (Deem et al. 2001). The focus of this special issue is to highlight that evolutionary thinking is an important and underused tool in management of wildlife disease.

Evolution is the mechanism by which wildlife adapt to novel circumstances, including pathogens. The dynamics of host–pathogen coevolution constitute one of the few examples of evolutionary rescue in vertebrates (e.g., Woodworth et al. 2005; Fenner 2010; Vander Wal et al. 2013b). Evolutionary rescue, however, represents an extreme case of adaptation among many evolutionary principles that are relevant to wildlife disease ecology and management (Table 1). These evolutionary principles are predicated on a number of measurable quantities (e.g., genetic variance, selection, gene flow, drift), which can be integrated in wildlife disease management. The realization that evolutionary processes can occur on ecological timescales (Carroll et al. 2007) further highlights the importance of evolutionarily enlightened management (*sensu* Ashley et al. 2003; see also Hendry et al. 2011; Lankau et al. 2011). For example, determining how the strength of selection varies with changing ecological conditions is critical for understanding the coevolution of host–pathogen dynamics. These ecological conditions include different host communities, multiple and novel pathogens, genotypes of a single pathogen, or changing environments (Vander Wal et al. 2014). Applying evolution to wildlife disease is therefore concerned with understanding how contemporary or historical selective pressures across ecological contexts shape current host–pathogen dynamics (Table 1).

**Table 1:** Relevant principles for evolutionarily enlightened wildlife disease management (adapted from Hendry et al. 2011; Lankau et al. 2011).

Evolutionary principle		Overview	Selected application to wildlife disease management	Reference within the special issue
Evolutionary history		An organism’s evolutionary history can inform on its origins and on potential constraints or facilitation acting on adaptation (i.e., probability of local adaptation).	Identifying cryptic pathogens and assessing their phylogeny.	Harrigan et al. (2014);
			Current distribution of phenotypes is typically a consequence of historical selective pressures.	Miller et al. (2014)
			Determining constraints on adaptation of pathogen virulence or host life-history traits, resistance, or tolerance.	
			Recognizing maladaptive behavioral responses that might predispose individuals to disease.	
Variation	Genetic variation	*Neutral genetic variation* (i.e., not under selection) acts as markers to determine population structure.	Gene flow can be indicative of population structure (e.g., spatial). Variation in neutral markers helps quantify landscape-level routes or barriers to disease spread. Alternately, if disease is locally isolated, similar information can be used to inhibit movement of individuals among subpopulations.	Rioux Paquette et al. (2014)
				Benavides et al. (2014)
			Determine local social structure or the demography of dispersal; both have implications for pathogen transmission.	
			Fostering connectivity can increase standing genetic variation and improve the probability that beneficial adaptations to spread to neighboring populations. Conversely, high gene flow can also inhibit local adaptation if selective pressures vary among subpopulations.	
		Underlying the expression of phenotypes is *additive genetic (co)variance*.	Response to selection requires additive genetic variation and should thus influence a trait potential to adapt.	Miller et al. (2014)
		*Functional genetic variation* (candidate gene)	Realistic assessments of host–pathogen evolutionary potential require an understanding of the stability of traits additive genetic (co)variance across ecological contexts.	
	Genotype-by-environment interactions	Plasticity is the individual response of a trait to different environmental contexts. Differential trait expression may thus occur from genotypes interacting with variable environments. This interaction can result in local adaptation.	Host–pathogen dynamics may vary across ecological contexts as a result of plasticity in trait expression of either host or pathogen or both.	Echaubard et al. (2014)
			Management practices may be suitable in one environment and unsuitable in an alternate environment.	
			Genotype × environment (or genotype × genotype) interactions may foster local adaptations in resistance or tolerance.	
	Phenotypic variation	Phenotypes govern how organisms (pathogen or host) interact with their environment.	Quantifying phenotypic variation (e.g., pathogen virulence, host immunity) establishes a baseline understanding of the variance upon which selection can act.	Lagagneux et al. (2014)
				Miller et al. (2014)
			Individual heterogeneity in behavior (e.g., ‘super-spreaders’), condition, etc., modulates disease spread and susceptibility.	
Selection		Selective pressures act through differential effects on reproductive success and survival (i.e., fecundity and viability selection) and can cause the mean and variance of a phenotype to vary across generations.	Pathogens can act as agents of selection.	Lagagneux et al. (2014)
			Management practices may select for individuals that have developed resistance or have high genetic diversity, and, consequently, may be more likely to develop resistance/immunity from standing genetic variation.	Simon et al. (2014)
			Policy or management that minimizes alternate (i.e., nondisease) sources of mortality may mitigate negative selective effects of disease.	
			Selection may be an unintended consequence of management practices, e.g., increase virulence or resistance to vaccines.	
Ecoevolutionary dynamics		The role phenotypic/genetic change via evolution plays in affecting ecological processes (and *vice versa*), either through feedback loops or correlations.	Coevolution between host and parasite (e.g., earlier age of primiparity and decreased virulence). Including coevolutionary feedback loops between pathogen and host may improve characterization of infectious disease dynamics [e.g., antibiotic resistance (Rivas et al. 2013)]	______
			Evolutionary rescue as a function of coadaptation between host and pathogen.	

**Figure 1:**
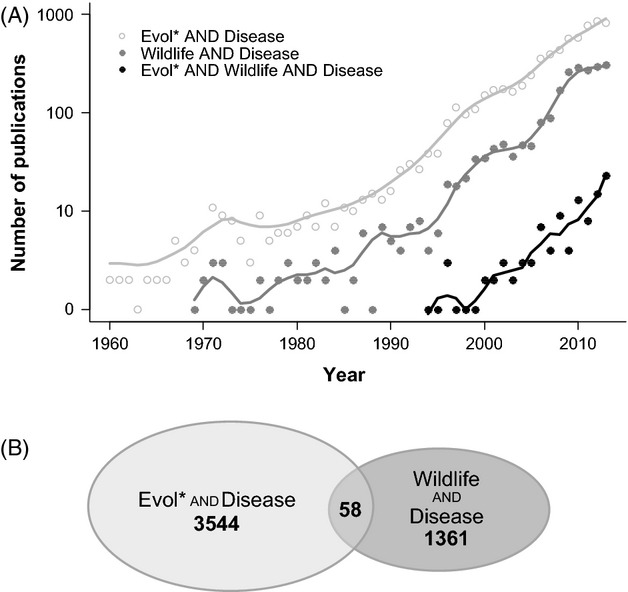
The number of publications on evolution and disease, wildlife and disease, and evolution, wildlife, and disease (A, 1960–2013) according to Scopus. We used the search terms (evol* AND disease; wildlife AND disease; evol* AND wildlife AND disease) as indicators and did not intend them to be comprehensive. (A) Positive trend in both the disciplines of disease evolution and wildlife disease exists. However, far fewer studies span the two (B, 2009–2013).

Yet, despite calls for increased integration of evolutionary concepts to wildlife disease ecology and management (Grenfell et al. 2004; Karesh et al. 2012), their application remains limited (Fig. 1). Although theory is becoming more prevalent in the wildlife disease literature, many contributions are descriptive natural history (≈50%; Joseph et al. 2013). Of those theory-driven contributions, the theories applied are predominantly ecological. Therefore, just as there is room to apply more ecological theory in wildlife disease (Tompkins et al. 2011), so too is there abundant space for the application of evolutionary theory. One example where evolutionary principles have been applied to wildlife disease to great effect is landscape genetics (Archie et al. 2009; Biek and Real 2010; Meentemeyer et al. 2012). These techniques have yielded important information on a number of host–pathogen systems (e.g., Sackett et al. 2011; Côté et al. 2012; Vander Wal et al. 2012; Altizer et al. 2013; Vander Wal et al. 2013a).

Other examples of applied evolution in the wild include (i) measuring selective forces shaping host–pathogen interactions (e.g., Jones et al. 2008b; Ujvari et al. 2014), (ii) incorporating genotype –, phenotype – and environment interactions into host–pathogen dynamics (Tack et al. 2012; Thrall et al. 2012), (iii) alleviating alternate selective pressures on species of concern to promote adaptation to novel pathogens, e.g., rodent removals and avian malaria (Kilpatrick 2006), (iv) facilitating or inhibiting gene flow to increase standing genetic variation or adaptation, respectively (Archie et al. 2009; Lankau et al. 2011), or (v) maintaining population sizes well below carrying capacity to minimize intraspecific competition, therefore increasing individual condition to invest in immunity (Kilpatrick 2006), or to capitalize on evolved mechanisms of transmission by promoting disease fade-out (Lloyd-Smith et al. 2005a). Management practices may also represent novel selective pressures that can hinder outcomes (Carroll 2011); for example, where pathogens develop resistance to vaccines (Gandon et al. 2003) or when selective culling of infected individuals results in increased virulence (Bolzoni and De Leo 2013).

Nevertheless, opportunities to apply evolutionary biology to wildlife disease ecology and management persist (Karesh et al. 2012). Because of continuing concerns of existing and emerging wildlife disease and their implications for population health, we argue that all tools available need to be employed to understand and manage disease in the wild. In an effort to bridge this gap, the objective of the special issue is to highlight invited case studies where evolutionary principles or tools were being applied to relevant wildlife disease issues.

## Main themes of the special issue

We tackle this challenge first by presenting a review synthesizing evolutionary applications to wildlife disease. Vander Wal et al. 2014 introduce an updated framework for understanding the complex ecological and evolutionary interactions that occur among systems that may have multiple competent hosts or agents in environments that are likely changing. Subsequent articles are primarily empirical and fit into one or more of three evolutionary principles: evolutionary history, phenotypic and genetic variation, and selection (Table 1).

Adaptability of organisms is influenced by their *evolutionary history*. For instance, evolutionary history, in combination with ecological heterogeneity, should shape taxonomic diversity of hosts and thus modulate the opportunity for parasites to exploit a wide or narrow breadth of hosts. This is shown by Harrigan et al. 2014 in the tropical Andes where a high biological richness and endemism translates into a rich diversity of avian hosts. Their analyses suggest that such high host diversity, in turn facilitates parasites (hemosporidia blood parasites) diversification and specialization.

*Neutral genetic variation* can also be used to understand how, when, and why a pathogen has spread in a population and can help managing future emergence of a disease. For example, in the case of cross-species transmission of bacterial pathogens, knowledge of the species involved in the transmission of a pathogen will determine whether control actions in one species may have subsequent effects on other host species. Identifying cross-species transmission in wild populations, however, is not a trivial task. Benavides et al. 2014 assess the ability of two types of genetic markers (variable number of tandem repeats and single-nucleotide polymorphisms) to distinguish between different transmission scenarios of cross-species transmission of bacterial pathogens. Their study suggests that even with whole genome sequences, unbiased estimates of cross-species transmission will be difficult when sampling is limited, mutation rates are low, or for pathogens that are recently introduced.

Coupling information from neutral markers with methods in landscape ecology to study dispersal indirectly can also illuminate patterns of disease spread and identify drivers or barriers of disease propagation (Blanchong et al. 2008; Côté et al. 2012). Anthropogenic change in land use can create new corridors and enhance disease spread. Rioux Paquette et al. 2014 tackle this question. They investigate the potential path of terrestrial rabies spread by raccoon (*Procyon lotor*) and skunk (*Mephitis mephitis*) using genetics analyses and spatially explicit models. Their models are constructed for a semipermeable landscape that, albeit affected by a gradient of intensifying agriculture, has few discrete barriers to host movement. The authors highlight habitat-specific corridors of gene flow that can be used to optimize current rabies vaccination programs.

*Genotype × environment* interactions can result in local adaptation when natural populations display large variation in susceptibility among hosts and in pathogenicity among virus strains. Such observations suggest that host and virus coevolve in response to each other, leading to coadaptation at a local scale, arising through genotype x genotype interactions. Local scale coadaptation is exemplified by the study of Echaubard et al. 2014 working with frog hosts and ranavirus strains under different temperature conditions. Specifically, they report that the impact of ranavirus was not only related to strain and host species identity but was also dependent on which genotypes were interacting with each other, suggesting potential coevolution in this system.

The importance of *functional genetic variation* is one of the topics explored by Miller et al. 2014 who perform an extensive literature review and present detailed cases studies to illustrate how infectious diseases affect salmonids. Importantly, they address how modern technologies (e.g., immune candidate gene, genome scan for loci quantitatively associated with disease, biotelemetry, and gene expression profiling) can improve the quality of ecological and evolutionary information needed to assess the impacts of disease processes in natural systems. They conclude by emphasizing how an approach including the cumulative and synergistic impacts of multiple stressors will help identify populations at greatest risk.

Disease can also affect the distribution of *phenotypic variation* available in wild populations and feedback on an organism’s interactions with their environment. As infection may reduce the physical condition and modulates immune responses of individuals, sick organisms may differ in their behavior, immune response, and susceptibility to predation to name only a few. For instance, Lagagneux et al. 2014 quantify the phenotypic variance in immune response varies during an epidemic. Miller et al. 2014 discuss the effects of phenotypic variation in condition based on predation in salmon. They found that predated salmon had much higher prevalence of pathogens compared to the mean population. This case study illustrates how pathogens can act in synergy with other ecological drivers and alter selective pressures.

*Selection* acts on individuals through differential survival or reproduction. Pathogens may exert a selective pressure on individuals indirectly through morbidity that contributes to reduced fecundity (devil facial tumor disease) or directly through mortality (e.g., avian cholera, *Pasteurella multocida*) or reproductive failure (e.g., brucellosis, *Brucella abortus*). In the process, selective pressures may also act on immunity. Here, Lagagneux et al. 2014 capitalize on an epidemic of avian cholera in a common eider (*Somateria mollissima*) colony to assess the strength, direction, and form of selection on immune traits. They also explore the potential for trade-offs between immune function and life-history traits using two fitness components. Despite high epidemic-induced mortality (Descamps et al. 2011), the authors find no clear evidence of selection on immune traits.

Environmental constraints can also represent important selective pressures. When those constraints are relaxed, it can facilitate host, vector, and pathogen spread. For example, when ranges are limited by temperature, climate warming may relax selective pressures (Altizer et al. 2013). Lyme disease is one currently expanding northward due to climate change. Here, Simon et al. 2014 predict the current distribution of *Borrelia burgdorferi* and model the risk of its expansion over a 50-year window factoring in the cooccurrence of the primary host and vector. They use climate niche models with landscape habitat models to understand which element (i.e., host, vector, or environment) is most likely to be the limiting factor for the spread of Lyme disease. The authors identify climate-driven range expansion, particularly of the white-footed mouse (*Peromyscus leucopus*), as the main factor predicting the northward movement of Lyme disease.

## Ethics of wildlife disease management

Humans actions are already recognized as an important selective force (Coltman et al. 2003; Darimont et al. 2009). Nonconsumptive forms of wildlife use, including research, can also have impacts on animals (McCallum and Hocking 2005; Vucetich and Nelson 2007; Paquet and Darimont 2010). As a result, there are ethical considerations underlying our actions when managing or researching wildlife disease. For instance, manipulating individuals can have unexpected and undesirable consequences on their health (Cattet et al. 2008). Often management actions involve more invasive techniques than manipulating individuals, including culling (Carter et al. 2007; Hallam and Mccracken 2011; White et al. 2011; Manjerovic et al. 2014). These actions are framed on a philosophy that focuses on prevention and eradication and discounts scenarios where novel pathogens may be providing an ecological or evolutionary service (e.g., as a selective pressure) or are ineradicable (see Carroll 2011 for an analogous discussion on invasive species). Even alternatives to culling, for example, vaccination programs, are not without serious ethical implications. One high profile case involved the impact of conservation actions on a rabies epidemic in African wild dogs (*Lycaon pictus*). Some researchers argued that rabies control operations contributed to the endangerment of the African wild dogs by accelerating the rate of population decline (Burrows 1992; Burrows et al. 1994)—a population decline that vaccinating individuals was intended to forestall or reverse. All packs that were vaccinated disappeared within a year while packs that were not manipulated persisted (Burrows 1992; Burrows et al. 1994; but see Creel 1992; Macdonald 1992). While the cause of death of these packs remains unknown, this is an example of the ethical considerations that managers and researchers face when responding to wildlife disease.

To close the special issue, Crozier and Schulte-Hostedde 2014 introduce that evolutionary and ecological science needs be science conducted ethically. These authors discuss the foundations of ethical research and provide a two-part framework that incorporates the best available science with sound ethical reasoning. This framework is aimed at helping decision makers avoid ethical pitfalls that commonly entrap wildlife disease managers.

## Challenges and future opportunities

An important challenge for applying evolutionary principles will be to overcome transdisciplinary boundaries that exist among the agencies that are collectively charged with managing wildlife disease. Managing wildlife disease is typically the responsibility of professionals with veterinary training and wildlife managers. These two groups oftentimes view disease at different scales: the former focused on individual health and the latter on population health (e.g., growth rates). Although management actions are undertaken at the population level, the processes such as selection and disease spread occur at the individual level. For example, individuals may vary in their susceptibility or propensity to spread disease (Lloyd-Smith et al. 2005b), with some individuals having a disproportionate effect on disease transmission through a population (Paull et al. 2012). This heterogeneity can arise from life-history, demographic, immunity, or behavioral factors (Cross et al. 2009). For example, socially isolated badgers (*Meles meles*) are more likely to spread bovine tuberculosis among badger sets; moreover, culling badgers further disrupt the social structure increasing tuberculosis prevalence (Carter et al. 2007; Weber et al. 2013). One main challenge is to overcome traditional boundaries between veterinarians, managers, and scientists to use approaches that span across scales to developed intervention strategies in managing wildlife disease.

To achieve this, lessons could be learned from the field of evolutionary medicine (Nesse and Stearns 2008; Stearns et al. 2010) where there is increasing effort to foster collaborations between physicians whom diagnose and treat patients with disease and evolutionary biologists whom understand the underlying (i.e., evolutionary) causes of disease [see, for example, controlling the spread of malaria (Mackinnon and Read 2004; Lyimo et al. 2013)]. One example includes imperfect vaccines selecting for increased virulence in pathogens (Gandon et al. 2003). Given the variation in immune response, vaccines can fail to create resistance in some individuals. This failure is associated with more virulent strains of a pathogen. Thus, these virulent strains are spread disproportionately throughout the susceptible population. Alternately, vaccination increases the costs to a pathogen without altering its benefits, resulting in increased virulence (Stearns 2012). Vaccines selecting for increased virulence have important evolutionary implications for malaria (Mackinnon et al. 2008) and human papilloma virus vaccination programs (Stearns 2012). While vaccines are often among the tools available to wildlife managers (Wobeser 2002); e.g., raccoon rabies variant (Rosatte et al. 2008), tuberculosis (Tompkins et al. 2013), brucellosis (Cross et al. 2013), their impermanence, and their role as selective agents are rarely considered.

## Closing remarks

Although this special issue presents a firm basis for progress, it only begins to touch on the potential value of evolutionarily enlightened wildlife disease management. The articles in the special issue present a core set of examples and highlight the progress that is being made in applying evolutionary principles to wildlife disease based on principles that fit into three categories: evolutionary history, phenotypic and genetic variation, and selection (Table 1). We contend that these, together with an ecoevolutionary dynamics framework, should be used as a roadmap for future application of evolution to wildlife disease management.
